# Correlation Between Serum and Urine Biomarkers and the Intensity of Acute Radiation Cystitis in Patients Treated With Radiation Therapy for Localized Prostate Cancer: Protocol for the Radiotoxicity Bladder Biomarkers (RABBIO) Study

**DOI:** 10.2196/38362

**Published:** 2023-01-10

**Authors:** Carole Helissey, Sophie Cavallero, Stanislas Mondot, Charles Parnot, Halima Yssaad, Selma Becherirat, Nathalie Guitard, Hélène Thery, Antoine Schernberg, Hugo Breitwiller, Cyrus Chargari, Sabine Francois

**Affiliations:** 1 Military Hospital Begin Saint-Mandé France; 2 Institut de Recherche Biomédicale des Armées Bretigny sur Orge France; 3 Paris-Saclay university Institut National de Recherche pour l'Agriculture Jouy-en-Josas France; 4 Cureety Dinan France

**Keywords:** radiation cystitis, radiotoxicity, urine, bladder, serum, quality of life, remote monitoring, biomarker, prostate, cancer, immunosorbent, urology, cytometry, protocol, telehealth, telemedicine, health platform, online platform, monitor, digital health, radiotherapy, radiation, risk, inflammation, inflammatory, sequencing, biopsy, biopsies, gene expression, protein, microbiology, cystitis, microbe, microbiota, RNA, proteomics, assay, algorithm, oncology, radiology, radiation therapy, prostate cancer, diagnostic, quality of life

## Abstract

**Background:**

Despite improvements in radiation techniques, pelvic radiotherapy is responsible for acute and delayed bladder adverse events, defined as radiation cystitis. The initial symptoms of bladder injury secondary to pelvic irradiation are likely to occur during treatment or within 3 months of radiotherapy in approximately 50% of irradiated patients, and have a significant impact on their quality of life. The pathophysiology of radiation cystitis is not well understood, particularly because of the risk of complications associated with access to bladder tissue after irradiation, which limits our ability to study this process and develop treatments.

**Objective:**

It is an original study combining digital data collection to monitor patients’ symptoms and biological markers during irradiation. The main objective of our study is to evaluate the correlation of biological biomarkers with the intensity of acute radiation cystitis and the quality of life of patients, assessed with the digital telemonitoring platform Cureety.

**Methods:**

Patients with intermediate-risk localized prostate cancer who are eligible for localized radiotherapy will be included. Inflammatory biomarkers will be analyzed in urine and blood samples before the start of radiotherapy and at weeks 4, 12, and 48 of irradiation, through quantitative methods such as a multiplex Luminex assay, flow cytometry, and enzyme-linked immunosorbent assay. We will also characterize the patients’ gut and urine microbiota composition using 16S ribosomal RNA sequencing technology. Between sample collection visits, patients will complete various questionnaires related to radiation cystitis symptoms (using the International Prostate Symptom Score), adverse events, and quality of life (using the Functional Assessment of Cancer Therapy–Prostate questionnaire), using the Cureety digital remote monitoring platform. Upon receipt of the questionnaires, an algorithm will process the information and classify patients in accordance with the severity of symptoms and adverse events reported on the basis of Common Terminology Criteria for Adverse Events and International Prostate Symptom Score standards. This will allow us to correlate levels of urinary, blood, and fecal biomarkers with the severity of acute radiation cystitis symptoms and patient-reported quality of life.

**Results:**

The study started in March 2022. We estimate a recruitment period of approximately 18 months, and the final results are expected in 2024.

**Conclusions:**

This prospective study is the first to explore the overexpression of inflammatory proteins in fluid biopsies from patients with symptoms of acute radiation cystitis. In addition, the 1-year follow-up after treatment will allow us to predict which patients are at risk of late radiation cystitis and to refer them for radioprotective treatment. The results of this study will allow us to develop strategies to limit radiation damage to the bladder and improve the quality of life of patients.

**Trial Registration:**

ClinicalTrials.gov NCT05246774; https://clinicaltrials.gov/ct2/show/NCT05246774?term=NCT05246774

**International Registered Report Identifier (IRRID):**

DERR1-10.2196/38362

## Introduction

### Background and Rationale

Prostate cancer is the leading cancer diagnosed in men in France, with 50,400 new cases and 8100 deaths in 2018. Improved diagnostic strategies and therapeutic management have led to a 3.7% reduction in mortality between 2010 and 2018, and the survival rate is 93% at 5 years and 80% at 10 years [1], resulting in improved overall survival. However, treatment-related adverse events can be significant and have an impact on adherence to treatment, frequency of hospitalization and associated costs, as well as on the health-related quality of life of patients [2].

Radiation therapy (including conventional radiation therapy, stereotactic body radiation therapy, and brachytherapy) is an important therapeutic technique in the management of pelvic cancers, including prostate cancer [3,4]. Despite improvements in radiation techniques, pelvic radiotherapy remains responsible for a number of acute and late bladder adverse events—these symptoms are grouped under “radiation cystitis.” In France, late radiation cystitis affects 9000 to 18,000 patients per year [5].

Early symptoms of radiation cystitis may occur during treatment and up to 3 months after the end of radiotherapy, with an estimated all grade incidence of nearly 50% after pelvic irradiation [5]. These side effects are characterized by frequent and urgent urination during day- and nighttime, a burning sensation during urination (irritative symptoms), and pain. Difficulty in urinating (obstructive symptoms) or, more rarely, blood in the urine (hematuria) may also be present [6]. More rarely, in 5%-10% of cases, complications appear later (more than 6 months after radiotherapy), whether or not they were preceded by early signs [5,7]. Late lesions involve blood vessel damage and fibrosis of the bladder wall, which may progress chronically and lead to bladder atrophy and even retraction in the most extreme cases [5]. Clinical signs vary depending on the dominant clinical form: cystalgia, pollakiuria, bladder hyperactivity, and isolated mictional disorders. In a classic clinical scenario, recurrent hematuria is a predominant symptom, occurring in abundance and with variable frequency, which can potentially progress to urinary retention with bladder clotting. The chronic and recurrent nature of hemorrhagic cystitis often has a considerable impact on the quality of life of patients. The most severe forms can lead to clot formation and acute urinary retention, which can be life threatening [5,7].

Although some factors have been identified, such as the dose received, fractionation and, comorbidities (eg, diabetes and tobacco smoking), the pathophysiology of radiation cystitis remains poorly studied, particularly because of the risks of complications arising from access to the bladder tissue post irradiation, thus limiting our knowledge of and therapies targeting this process*.*

### The Role of Immunity in the Pathomechanism of Radiation-Induced Injury

Immunity plays an important role in the mechanism of radiation-induced toxicity or inflammation [8,9]. During the repair process of radiation-induced injuries, inflammatory cells (macrophages, neutrophils, and lymphocytes) are recruited to the site of injury, which may contribute to late inflammatory tissue diseases through a continuous mechanism involving inflammation, hypoxia, and fibrosis [10]. The balance between M1 and M2 macrophages plays a central role in the fibrotic process, with a polarization toward M1 macrophages in a fibrotic situation [11,12]. Functional tests measuring the apoptosis of CD4^+^ and CD8^+^ T lymphocytes after irradiation found a significant association between these apoptotic lymphocytes and the risk of occurrence of late genitourinary toxicity [13].

The interstitial cystitis model is similar to the radiation cystitis model in terms of not only collagen accumulation but also symptoms. Patients with interstitial cystitis have very severe genitourinary pain, and many are diagnosed with depression and anxiety. A positive correlation between elevated urinary proinflammatory cytokines (interleukin [IL] 4 and macrophage-derived chemokines) and the severity of interstitial cystitis has been reported [14,15].

### Role of the Microbiota in the Pathomechanism of Radiation-Induced Lesions

Evidence of the protection of microbial communities, especially in the gastrointestinal tract, has led to investigations of the role of the human microbiota in patient health and well-being [16,17].

Radiation therapy to the prostate causes a disruption in the composition of the microbiota, which may promote gastrointestinal toxicities through altered gut barrier function and inflammation [18,19]. It has been shown that the gut microbiota of patients with pelvic cancers who have had radiotherapy is less diverse than that of patients who have not been irradiated [18]. Wang et al [19,20] reported that pelvic radiotherapy is associated with dysbiosis characterized by a decrease in α-diversity in favor of β-diversity. In order to reduce the digestive disorders induced by pelvic radiotherapy, an approach based on fiber supplementation in patients (partially hydrolyzed guar gum) has proven to be effective in reducing diarrhea induced by radiotherapy and has allowed the improvement of the bacterial load of the microbiota while improving nutritional status and the quality of life of patients with cancer [21]. However, the numbers considered in Bull et al’s [21] study were limited. Another study [22] also considered fecal microbiota transfer as a therapeutic solution to slow the spread of radiation-related symptoms—this approach showed encouraging signals for reducing the symptoms of hematuria and diarrhea.

Biomarkers based on the urinary microbiota may represent new diagnostic, prognostic, and therapeutic tools for functional disorders of the lower urinary tract. It has been shown that the urinary microbiota of patients with interstitial cystitis is less diverse than that of patients without these symptoms [20].

To date, no study has evaluated the modification of the urinary microbiota in patients with radiation cystitis. A better understanding of the impact of the urinary microbiota on the etiopathogenesis of urological disorders may help optimize medical management.

It is also important to note that the microbiota of different organs are linked. It is known that the gut microbiota is capable of changing its microbial composition, and these changes can affect the urinary microbiota [21].

### Main Objective

The main objective of this study is to determine inflammatory and remodeling markers involved in the occurrence of early (<3 months) radiation cystitis in patients with localized prostate cancer.

### Secondary Objectives

Our secondary objectives are to determine biological markers of the severity of early radiation cystitis and to describe changes in urinary and fecal microbiota in accordance with the severity of early radiation cystitis.

### Exploratory Objectives

Our exploratory objectives are to describe the biological markers of severity of late radiation cystitis (>6 months) and to describe the lipidome and metabolome changes in accordance with the precocity of radiation cystitis.

## Methods

### Study Design

The Radiotoxicity Bladder Biomarkers (RABBIO) study is an interventional, prospective, single-arm, exploratory study aimed to identify factors potentially related to radiation-induced bladder toxicity in patients with localized prostate cancer treated with radiotherapy. This study will be conducted at the Bégin Military Teaching Hospital (Saint-Mandé, France).

### Ethical Considerations

The study has been validated by national ethics committees (unique protocol ID number 2021-A03196-35; favorable opinion of the South Mediterranean Committee for the Protection of Persons I 03/02/2022) and the French Data Protection Agency. The study is registered on ClinicalTrials.gov (NCT05246774). The survey complies with the tenets of the Declaration of Helsinki. All patients will be informed that the data collected may be used for research purposes, and will be asked to provide their written consent.

### Patient Population

The eligibility criteria for the RABBIO study are listed in Textbox 1.

**Inclusion and exclusion criteria.**

**Inclusion criteria:**

- Collection of signed informed consent forms prior to participation in the study
- Patients are aged ≥18 years at the time of selection
- Histologically confirmed adenocarcinoma of the prostate is present
- Localized adenocarcinoma of the prostate according to the D'Amico classification is present
- Patients are eligible for external radiotherapy or brachytherapy
- Patients are affiliated to a social security scheme
- Patients are able to communicate well, understand, and comply with the requirements of the study according to the physician investigator
- Patient has a smartphone or computer to use the Cureety platform

**Exclusion criteria:**

- Patients have advanced or metastatic prostate cancer
- Patients are receiving preirradiation hormone therapy
- Patients have bladder or urethral cancer or a history of cancer
- Previous urinary tract surgery (bladder augmentation or cystectomy)
- Patients are participating in an interventional clinical study
- Patients have a history of pelvic irradiation

### Participants’ Calendar

Early symptoms of radiation cystitis are likely to occur during treatment or within 3 months of radiotherapy in approximately half of the patients [7]. Therefore, we decided to follow the early manifestations of radiation-induced bladder toxicity for 3 months (weeks 1 to 12) and to attempt to identify biomarkers that are potentially related to acute radiation cystitis symptomatology.

Late manifestations of bladder damage secondary to pelvic irradiation may occur after a minimum of 3 months or even several years after the end of irradiation [4,6]. It has been established that the average delay in the occurrence of these complications is 2 years [6]. However, the pathophysiological changes between the end of radiation and the occurrence of late complications are not known. In this study, we aim to, as an exploratory objective, determine biomarkers that may be predictive of late radiation cystitis by quantifying biomarkers at week 52.

**Figure 1:**
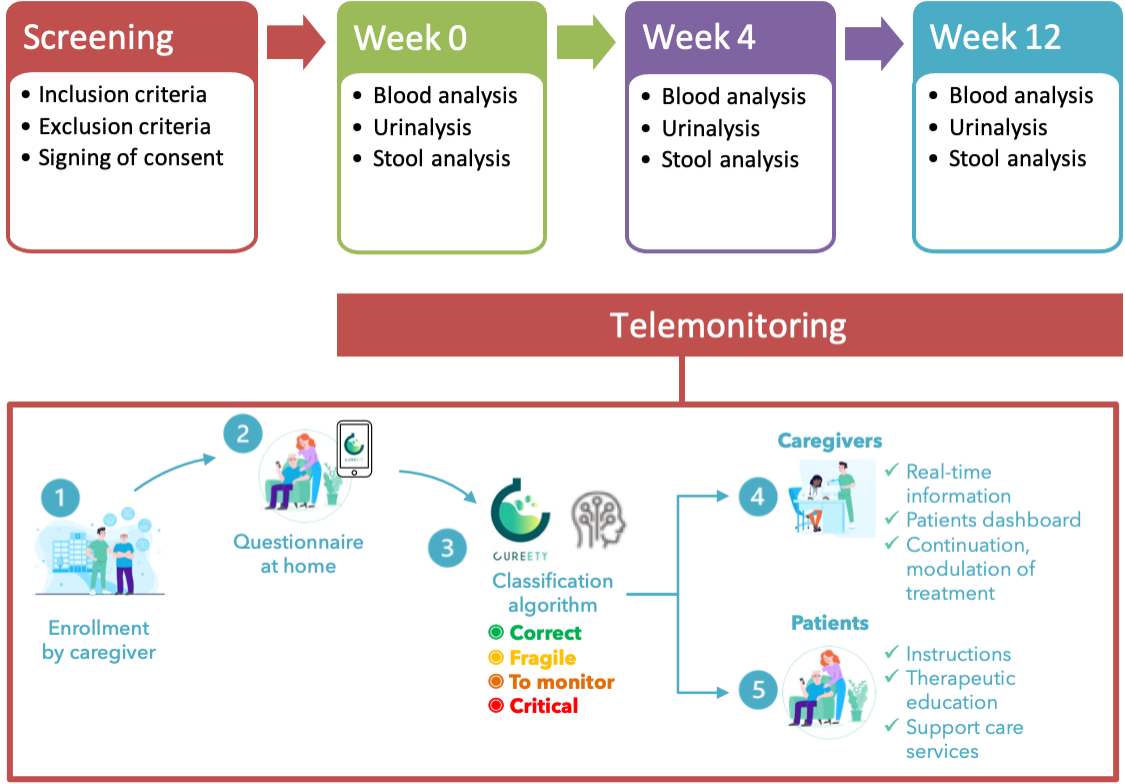
Study design.

The design of RABBIO study is shown in Figure 1.

### Data Collection

#### Clinical Data

##### Demographics and Disease Characteristics

Demographic data and cancer characteristics (localized or biologically relapsed prostate cancer, stage of disease, radiation regimen, concomitant treatments, and comorbidities) of the patients will be collected.

##### Clinical Examination

Clinical examination at each visit will include performance index (performance status), weight, blood pressure, heart rate, and oxygen saturation. 

##### Monitoring of Radiotherapy Adverse Events and Patient-Reported Quality of Life Using the Cureety Platform

The Cureety platform will allow remote monitoring of urinary symptoms reported by patients in accordance with the CTCAE (Common Terminology Criteria for Adverse Events) guidelines [22] (Multimedia Appendix 1). 

Patients will complete the Pelvic Radiation Adverse Events Questionnaire at the inclusion visit, then once a week for 3 months (weeks 1 to 12), and then at the end-of-study visit (week 52). The questionnaire includes the following items: fatigue, nausea or vomiting, pain, hematuria, frequency of urination (pollakiuria), urinary burning, diarrhea, fecal incontinence, urinary leakage, blood in the stool (rectorrhagia), constipation, weight loss, and dysuria.

**Figure 2:**
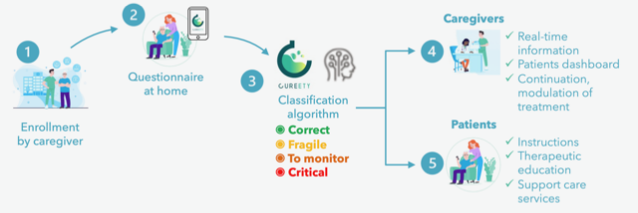
Monitoring of radiotherapy adverse events and patient-reported quality of life using the Cureety app.

On receipt of the questionnaires on the adverse effects of radiation, an artificial intelligence algorithm will classify patients into 1 of 4 states: correct condition (green), fragile state (yellow), condition to monitor (orange), and critical condition (red; Figure 2). The patient will receive therapeutic advice depending on the severity of the symptoms. If the patient’s condition changes to orange (watch) or red (critical), rapid management of the patient will be activated by the health care team (Figure 2).

##### International Prostate Symptom Score

The International Prostate Symptom Score (IPSS) [23] is a structured, validated, self-administered questionnaire that assesses lower urinary tract voiding disorders. The questions cover the following items: incomplete emptying of the bladder, frequency of micturition, intermittent micturition (stopping and restarting the stream), urgent micturition (feeling of “urgency”), weak stream, effort to urinate (forcing or pushing), and nocturia (Multimedia Appendix 1).

##### The Functional Assessment of Cancer Therapy–Prostate Questionnaire

The Functional Assessment of Cancer Therapy–Prostate (FACT-P) questionnaire [24] is a prostate cancer–specific, self-administered questionnaire that assesses weight loss, appetite, pain, physical comfort, urinary, and sexual and bowel function in 12 items. The score ranges from 0 to 156, with higher scores reflecting a better quality of life.

##### The International Physical Activity Questionnaire

The International Physical Activity Questionnaire (IPAQ) [25] is a 7-item questionnaire that assesses overall physical activity and sedentary time over the past 7 days. The questionnaire assesses intense or moderate walking activity, as well as time spent sitting (sedentary), whether during leisure activities, at work, in daily life, or during transport. The questionnaire classifies the subject in accordance with 3 levels of activity: inactive, moderate, and high [25].

The IPSS, FACT-P and IPAQ questionnaires will be completed by the patient via the Cureety platform at inclusion and at the visits at weeks 4, 12, and 52.

### Biological Data

#### Overview

Nearly 2 dozen studies have defined urinary biomarkers indicative of visceral disorders. Three main categories of markers have been studied: proteins involved in epithelial cell growth, mediators of inflammation, and neurotrophins. All marker levels were significantly altered in patients with interstitial cystitis, but antiproliferative factor, epidermal growth factor, and heparin-binding epidermal growth factor were the markers with the most promising results [26,27]. For radiation cystitis, the 2 studies by Zwaans et al [9,28] are the only ones to report elevated urinary levels of plasminogen activator inhibitor 1, the matrix metalloproteinases tissue inhibitor of matrix metalloproteinase 1 and 2, hepatocyte growth factor, VEGF-A, and placental growth factor. Based on the biomarkers reported in the literature, we chose to assess variations in the expression of 33 serum and urine biomarkers [9,28].

Biomarkers of urinary and intestinal microbiota may be prognostic of radiation-induced functional disorders [18-20]. Thus, we chose to assess changes in the composition of the urinary and fecal microbiota before, during, and after radiotherapy.

Physical activity, as determined on the basis of muscle contraction and the oxygen consumption it generates, can induce changes in the gut microbiota profile, helping to explain the health benefits of exercise. Researchers have identified differences in the composition of the gut microbiota of elite athletes in comparison with those of sedentary individuals [29]. A recent study has shown that the composition of the microbiota also differs in accordance with the sport practiced [30].

The collected samples will be analyzed for circulating biomarkers and microbiota composition.

#### Inflammatory and Remodeling Biomarkers

Overall, 6 mL of blood and 5 mL of urine per patient per visit will be used for the analysis of the following 33 biomarkers: inflammatory biomarkers (including macrophage migration inhibitory factor; cytokines IL-1α, IL-1β, IL-4, IL-6, IL-7, IL-8, IL-10, IL-13, and IL-17α; macrophagic inflammatory protein 1α; TNF-α; vascular cell adhesion molecule 1; intercellular adhesion molecule 1; chemotactic cytokines including monocyte chemoattractant protein 1 and 3 and regulated on activation, normal T-expressed, and secreted; the C-X-C motif chemokine ligand 10; the M1:M2 macrophage ratio; CD4^+^ and CD8^+^ T cells; and CRP) and biomarkers of remodeling (including plasminogen activator inhibitor 1, metalloproteinases such as matrix metalloproteinase 9, matrix metalloproteinase inhibitors including tissue inhibitor of matrix metalloproteinase 1 and 2, hepatocyte growth factor, placental growth factor, VEGF, epidermal growth factor, heparin-binding epidermal growth factor, nerve tissue growth factor, and glycoprotein GP51).

#### Urinary and Fecal Microbiota

The advent of molecular biology and high-throughput sequencing has revealed the diversity of urinary and fecal microbiota and led to a better understanding of these ecosystems. Over the past 2 decades, shotgun metagenomic and 16S ribosomal RNA sequencing approaches have been widely used to determine the composition of these microbiota.

#### Lipidome and Metabolome

This involves the analysis of metabolites (small molecules) and lipids in the blood and urine before, during, and after radiation therapy. The identity and quantity of the different metabolites and lipids depend on several factors such as available nutrients, environmental stimuli, or physiological state. Metabolome and lipidome analyses help elucidate the influence of radiotherapy on the expressed phenotype and metabolism of the patient. A GC 7890B gas chromatography system (Agilent) coupled to a MS 7010 triple quadrupole mass spectrometer will help identify lipid and metabolic proinflammatory mediator profiles in the serum and urine of patients.

### Statistical Analysis

As this is a pilot exploratory study, the sample size was not based on a statistical argument. As the variability and evolution of biomarkers over time and the history of the disease are not known, and in order to explore the links between biomarkers and the occurrence of radiation cystitis, based on the hypothesis that half of the patients initially included will develop cystitis, a sample size of 60 individuals—including 30 individuals with cystitis and 30 individuals without cystitis—seems acceptable. Statistical analysis will be carried out using SAS (version 9.4; SAS Institute Inc) and R (The R Foundation) [31].

Basic statistics will be used for continuous variables n, missing n (if applicable), mean, type of deviation, median, first and third quartiles, and minimum and maximum values, and for categorical variables, we will use frequency and percentage values.

The type I error (α, 2-sided) will be 5%. The type II error (β) will be 20%; that is, a power (1-β) of 80% will be considered.

### End Points and Evaluation

The primary end point will indicate variations in the expression of the 33 inflammatory and remodeling biomarkers assessed using the MILLIPLEX MAP technique for the analysis of circulating markers and flow cytometry for the analysis of the immune population at the 4th and 12th weeks after the start of irradiation. 

The secondary endpoints will indicate symptoms and the severity of early radiation cystitis through electronic reporting of patient-reported outcomes–CTCAE self-assessment questionnaires at inclusion and once a week during weeks 1 to 12. We will also be interested in voiding disorders, quality of life, and physical activity assessed using the IPSS, FACT-P, and IPAQ self-assessment questionnaires at inclusion and at weeks 4 and 12. Furthermore, we will characterize the composition of the urinary and fecal microbiota via 16S ribosomal RNA sequencing at baseline and at weeks 4 and 12.

Finally, we shall explore the eligibility criteria, and then at week 52, the symptoms and severity of cystitis will be assessed through electronic reporting of patient-reported outcomes–CTCAE self-assessment questionnaires, and voiding disorders, quality of life, and physical activity will be assessed with the IPSS, FACT-P, and IPAQ self-assessment questionnaires, and then the composition of the urinary and fecal microbiota will be characterized through 16S ribosomal RNA sequencing. We will analyze serum and urine lipidome and metabolomes at weeks 4, 12, and 52.

## Results

The RAABIO study started in March 2022. It is an original study combining digital data collection to monitor patients' symptoms and biological markers during irradiation. We estimate a recruitment period of approximately 18 months. The final results are expected in 2024.

## Discussion

The quality of life of long-surviving patients is a goal of their care. Minimizing the impact of our treatments remains very challenging. This study is expected to improve our knowledge of the pathophysiology of radiation cystitis and its impact on the quality of life of our patients.

Radiation cystitis—which is characterized by hematuria, inflammation, and partly by fibrosis—has a strong impact on the daily life of our patients. Telemonitoring allows the recording of patients' experiences and the assessment of the impact of side effects on his quality of life. Collecting data from the patient (patient-reported outcomes) help to correct the discrepancy in the severity of the side effects when reported by the clinician or by the patient. Remote monitoring has been shown to provide high-quality care and has the potential to significantly improve patient care.

An increasing number of pathologies or their treatments, including radiation therapy of the prostate, now report alterations of the host-microbiome symbiosis [32,33]. In addition to remote monitoring patients’ experiences, disorders involving the gastrointestinal area could be assessed by monitoring host-microbiome interactions using wireless ingestible capsules technologies as described recently by Miley et al [34]. These emerging technologies could be useful in tailoring daily treatments and helping patients reduce the side effects of radiation.

This prospective study is the first to explore the overexpression of inflammatory proteins in fluid biopsies from patients with symptoms of acute radiation cystitis. In addition, the 1-year follow-up after treatment will allow us to predict which patients are at risk of late radiation cystitis and to guide them toward radioprotective treatment. The RABBIO study will provide a better understanding of the pathophysiology of radiation-induced cystitis, along with data on the kinetics of these biomarkers. The results of this study will allow us to develop strategies to limit radiation damage to the bladder and improve the quality of life of patients.

## Appendix

Multimedia Appendix 1 The Common Terminology Criteria for Adverse Events (version 5.0) questionnaire for classification of noninfectious cystitis and the International Prostate Symptom Score for the severity of prostate cancer symptoms.

## Abbreviations

CTCAE: Common Terminology Criteria for Adverse Events
FACT-P: Functional Assessment of Cancer Therapy–Prostate
IL: interleukin
IPAQ: International Physical Activity Questionnaire
IPSS: International Prostate Symptom Score
RABBIO: Radiotoxicity Bladder Biomarkers

## Data Availability

The data sets generated or analyzed during this study are available from the corresponding author on reasonable request.

## References

[1] Le cancer de la prostate. Institute National du Cancer.

[2] Taylor JM, Chen VE, Miller RC, Greenberger BA (2020). The impact of prostate cancer treatment on quality of life: a narrative review with a focus on randomized data. RRU.

[3] (2019). American Cancer Society.

[4] Cancers uronéphrologiques. Institute National du Cancer.

[5] Helissey C, Cavallero S, Brossard C, Dusaud M, Chargari C, François S (2020). Chronic inflammation and radiation-induced cystitis: molecular background and therapeutic perspectives. Cells.

[6] Rigaud J, Le Normand L, Labat J- (2010). Cystite radique. Pelv Perineol.

[7] Goucher G, Saad F, Lukka H, Kapoor A (2019). Canadian Urological Association best practice report: diagnosis and management of radiation-induced hemorrhagic cystitis. Can Urol Assoc J.

[8] Miller KD, Nogueira L, Mariotto AB, Rowland JH, Yabroff KR, Alfano CM, Jemal A, Kramer JL, Siegel RL (2019). Cancer treatment and survivorship statistics, 2019. CA Cancer J Clin.

[9] Zwaans BMM, Nicolai HE, Chancellor MB, Lamb LE (2020). Prostate cancer survivors with symptoms of radiation cystitis have elevated fibrotic and vascular proteins in urine. PLoS One.

[10] Chargari C, Supiot S, Hennequin C, Chapel A, Simon J (2020). [Treatment of radiation-induced late effects: What's new?]. Cancer Radiother.

[11] Mukherjee D, Coates PJ, Lorimore SA, Wright EG (2014). Responses to ionizing radiation mediated by inflammatory mechanisms. J Pathol.

[12] Meziani L, Deutsch E, Mondini M (2018). Macrophages in radiation injury: a new therapeutic target. OncoImmunology.

[13] Foro P, Algara M, Lozano J, Rodriguez N, Sanz X, Torres E, Carles J, Reig A, Membrive I, Quera J, Fernandez-Velilla E, Pera O, Lacruz M, Bellosillo B (2014). Relationship between radiation-induced apoptosis of T lymphocytes and chronic toxicity in patients with prostate cancer treated by radiation therapy: a prospective study. Int J Radiat Oncol Biol Phys.

[14] Kim J, Kim WT, Kim W (2020). Advances in urinary biomarker discovery in urological research. Investig Clin Urol.

[15] Vera PL, Preston DM, Moldwin RM, Erickson DR, Mowlazadeh B, Ma F, Kouzoukas DE, Meyer-Siegler KL, Fall M (2018). Elevated urine levels of macrophage migration inhibitory factor in inflammatory bladder conditions: a potential biomarker for a subgroup of interstitial cystitis/bladder pain syndrome patients. Urology.

[16] Silva MJB, Carneiro MBH, dos Anjos Pultz B, Pereira Silva D, Lopes MEDM, dos Santos LM (2015). The multifaceted role of commensal microbiota in homeostasis and gastrointestinal diseases. J Immunol Res.

[17] Althani AA, Marei HE, Hamdi WS, Nasrallah GK, El Zowalaty ME, Al Khodor S, Al-Asmakh M, Abdel-Aziz H, Cenciarelli C (2016). Human microbiome and its association with health and diseases. J Cell Physiol.

[18] Reis Ferreira M, Andreyev H, Mohammed K, Truelove L, Gowan S, Li J, Gulliford S, Marchesi J, Dearnaley D (2019). Microbiota- and radiotherapy-induced gastrointestinal side-effects (MARS) study: a large pilot study of the microbiome in acute and late-radiation enteropathy. Clin Cancer Res.

[19] Wang Z, Wang Q, Wang X, Zhu L, Chen J, Zhang B, Chen Y, Yuan Z (2019). Gut microbial dysbiosis is associated with development and progression of radiation enteritis during pelvic radiotherapy. J Cell Mol Med.

[20] Wang A, Ling Z, Yang Z, Kiela PR, Wang T, Wang C, Cao L, Geng F, Shen M, Ran X, Su Y, Cheng T, Wang J (2015). Gut microbial dysbiosis may predict diarrhea and fatigue in patients undergoing pelvic cancer radiotherapy: a pilot study. PLoS One.

[21] Bull C, Devarakonda S, Ahlin R (2021). Role of dietary fiber in safeguarding intestinal health after pelvic radiotherapy. Curr Opin Support Palliat Care.

[22] Zheng Y, He X, Xia HH, Yuan Y, Xie W, Cai J, Xu J, Wu L (2020). Multi-donor multi-course faecal microbiota transplantation relieves the symptoms of chronic hemorrhagic radiation proctitis: A case report. Medicine (Baltimore).

[23] International prostate symptom score (IPSS). Royal United Hospitals Bath. NHS Foundation Trust.

[24] FACT-P. Functional Assessment of Cancer Therapy – Prostate. For patients with prostate cancer. FACIT.org.

[25] Craig CL, Marshall AL, Sjöström M, Bauman AE, Booth ML, Ainsworth BE, Pratt M, Ekelund U, Yngve A, Sallis JF, Oja P (2003). International physical activity questionnaire: 12-country reliability and validity. Med Sci Sports Exerc.

[26] Kuo H (2014). Potential urine and serum biomarkers for patients with bladder pain syndrome/interstitial cystitis. Int J Urol.

[27] Peyronnet B, Bendavid C, Manunta A, Damphousse M, Cheensse C, Brochard C, Castel-Lacanal E, Siproudhis L, Bensalah K, Gamé X (2015). [The role of urinary markers in the assessment and follow-up of lower urinary tract disorders: a literature review]. Prog Urol.

[28] Zwaans BM, Bartolone SN, Chancellor MB, Nicolai HE, Lamb LE (2018). Altered angiogenic growth factors in urine of prostate cancer survivors with radiation history and radiation cystitis. Urology.

[29] Rankin A, O’Donovan C, Madigan SM, O'Sullivan O, Cotter PD (2017). ‘Microbes in sport’ – The potential role of the gut microbiota in athlete health and performance. Br J Sports Med.

[30] O'Donovan CM, Madigan SM, Garcia-Perez I, Rankin A, O' Sullivan O, Cotter PD (2020). Distinct microbiome composition and metabolome exists across subgroups of elite Irish athletes. J Sci Med Sport.

[31] The R Project for Statistical Computing.

[32] van de Guchte M, Blottière HM, Doré J (2018). Humans as holobionts: implications for prevention and therapy. Microbiome.

[33] Lam KN, Alexander M, Turnbaugh PJ (2019). Precision medicine goes microscopic: engineering the microbiome to improve drug outcomes. Cell Host Microbe.

[34] Miley D, Machado LB, Condo C, Jergens AE, Yoon K, Pandey S (2021). Video capsule endoscopy and ingestible electronics: emerging trends in sensors, circuits, materials, telemetry, optics, and rapid reading software. Adv Device Instrum.

