# Supplementary material for: Correlation Between Serum and Urine Biomarkers and the Intensity of Acute Radiation Cystitis in Patients Treated With Radiation Therapy for Localized Prostate Cancer: Protocol for the Radiotoxicity Bladder Biomarkers (RABBIO) Study
Source: JMIR Res Protoc. 2023 Jan 10;12:e38362. doi: 10.2196/38362 (PMC9874987; doi:10.2196/38362)
Supplement: Multimedia Appendix 1 [file resprot_v12i1e38362_app1.docx]

| Grade 0: no symptoms |
| --- |
| Grade 1: Mild symptomatic, microscopic hematuria, minimal increase in frequency, dysuria, or nocturia, incontinence. |
| Grade 2: Pollakiuria with dysuria; macroscopic hematuria, moderate hematuria, urinary catheter placement or bladder irrigation indicated, limited instrumental activities of daily living (IADL). |
| Grade 3: Gross hematuria, transfusion, intravenous analgesics, indication for bladder irrigation. |
| Grade 4: Catastrophic bleeding; major indication for intervention. |
| Year 5: Death |

Supplementary Text Box 1. CTCAE v5.0 (Common Terminology Criteria for Adverse Events version 5.0) classification for non-infectious cystitis

| Score of 0 - 7: Not very symptomatic |
| --- |
| Score of 8 - 19: Moderately symptomatic |
| Score of 20 - 35: Severe symptoms |

Supplementary Text Box 2. International Prostate Symptom Score. Each question has a score from 1 to 5, for a total of 35 points maximum. The total of the 7 items gives the international score for prostate symptoms in terms of severity
